# Validity and Stability of the Decisional Balance for Sun Protection Inventory

**DOI:** 10.1155/2014/190541

**Published:** 2014-12-07

**Authors:** Hui-Qing Yin, Joseph S. Rossi, Colleen A. Redding, Andrea L. Paiva, Steven F. Babbin, Wayne F. Velicer

**Affiliations:** Cancer Prevention Research Center, University of Rhode Island, 130 Flagg Road, Kingston, RI 02881, USA

## Abstract

The 8-item Decisional Balance for sun protection inventory (SunDB) assesses the relative importance of the perceived advantages (Pros) and disadvantages (Cons) of sun protective behaviors. This study examined the psychometric properties of the SunDB measure, including invariance of the measurement model, in a population-based sample of *N* = 1336 adults. Confirmatory factor analyses supported the theoretically based 2-factor (Pros, Cons) model, with high internal consistencies for each subscale (*α* ≥ .70). Multiple-sample CFA established that this factor pattern was invariant across multiple population subgroups, including gender, racial identity, age, education level, and stage of change subgroups. Multivariate analysis by stage of change replicated expected patterns for SunDB (Pros *η* ^2^ = .15, Cons *η* ^2^ = .02). These results demonstrate the internal and external validity and measurement stability of the SunDB instrument in adults, supporting its use in research and intervention.

## 1. Introduction

Skin cancer is a major public health concern. Melanoma is the most serious form of skin cancer and accounts for the majority of skin cancer deaths. The American Cancer Society estimates there will be more than 76,000 new cases of melanoma diagnosed in 2014 in the United States. Nonmelanoma skin cancers are typically nonfatal but much more prevalent; in 2006, approximately 3.5 million people in the United States were diagnosed with these malignancies, and more than 2 million were treated [1]. The incidence rates for both types of skin cancers have been increasing [1, 2]. Skin cancers lead to substantial direct medical care costs and significant indirect costs associated with premature mortality and morbidity [3, 4]. Preventing all skin cancers is both important and possible by adopting habitual sun protective behaviors such as reducing sun exposure and using sunscreen [5].

Interventions for increasing sun protection behaviors using tailored health communications based on the transtheoretical model of behavior change have been developed and implemented and have demonstrated significant impacts in numerous applications [6–9]. The transtheoretical model (TTM) [10–13] is an integrative model of intentional behavior change underlying numerous effective interventions. Empirically based tailoring is especially relevant in population-based interventions when not everyone is prepared to immediately change their risk behavior(s). Decisional Balance is one of the core constructs integrated within the TTM framework. Based initially on the work of Janis and Mann [14], Decisional Balance reflects the cognitive and motivational shifts in decision making as an individual weighs the relative importance of the Pros and Cons of changing the behavior in question [15]. The theoretical relationship between Decisional Balance and transitions across the stages of change (i.e., readiness to change the problem behavior) has been well documented across a variety of health behaviors [16, 17], and therefore incorporated into intervention programs. Appropriately operationalizing theoretical constructs into psychometrically sound measures is critical for testing and implementing a theoretical model. The Decisional Balance for sun protection inventory has been used in a number of applications [7–9]; however, no published study has evaluated the psychometric properties of this measure.

The aim of this study was to assess the psychometric properties of the Decisional Balance for sun protection instrument, including confirming the factorial invariance of the measure across population subgroups. Factorial invariance is central to establishing the validity of a measure, as it indicates whether a set of items measures the same theoretical constructs across subgroups, allowing legitimate comparisons between groups on the measure of interest [18]. Three levels of factorial invariance were assessed in sequential order, with increasing levels of restrictiveness or equality constraints on parameters in the model. Configural invariance is an unconstrained model, in which the number of factors and the specific items associated with each factor are assumed to be the same across comparison groups. Pattern identity invariance is the next level of invariance and requires factor loadings for the same items to be equal across groups. Strong factorial invariance is the most restrictive of these three levels and requires factor loadings and error variances to be the same across groups [18, 19]. Meaningful group comparisons can be assumed when a measure has demonstrated strong factorial invariance.

The present study involved secondary analysis of baseline data for a large population-based sample of adults enrolled in a randomized TTM-tailored intervention study targeting sun protection and exercise behaviors. The theoretical structural model of the Decisional Balance for sun protection measure was assessed. The factorial invariance of the measurement model was then tested across population subgroups defined by gender, racial identity, age, education, untanned skin color (a proxy for sun reactivity), and stage of change for sun protection. Mean differences for Decisional Balance by stages of change were also examined to see if expected patterns based on TTM predictions would replicate. Confirming measurement structure and stability, and the functional relationship between the Decisional Balance constructs and stages of change, provides a necessary empirical foundation for TTM-tailored interventions.

## 2. Method

### 2.1. Sample

Participants were population-based adults (age range 18–75 years, 88% white, and 63% female) from across the United States enrolled in a recent intervention study for exercise and sun protection based on the TTM [20]. Participants in the sample were all “at risk” for both behaviors at baseline based on public health criteria. Participants were identified to be “at risk” for sun exposure if they reported not* consistently* (i) using sunscreens with a sun protection factor (SPF) of 15 or more, (ii) wearing protective clothing, and (iii) avoiding or limiting exposure to the sun during the midday hours. All procedures were approved by the Institutional Review Board at the University of Rhode Island, and participants understood and consented to their voluntary involvement in the study. Data for these analyses were drawn from the baseline assessment collected from April 2009 through September 2011.

### 2.2. Sun Protection Decisional Balance Instrument

**Figure 1:**
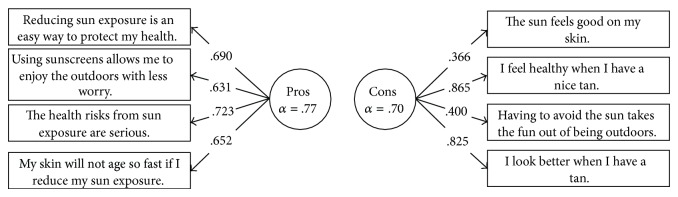
Measurement model for uncorrelated Pros and Cons of sun protection with standardized parameter estimates for full sample (*N* = 1336).

The 8-item Decisional Balance instrument assessed the perceived advantages (Pros) and disadvantages (Cons) of sun protection. This measure consists of two subscales: a four-item Pros scale and a four-item Cons scale and was developed and successfully employed in a number of previous randomized intervention trials [7–9, 21]. The instrument asked respondents to rate how important each item was in deciding whether or not to protect themselves from too much sun exposure on a 5-point Likert scale from 1 (not important) to 5 (extremely important). The theoretical measurement model for Decisional Balance with two latent Pros and Cons factors (see Figure 1) was assessed for the invariance analyses with this sample.

### 2.3. Data Analyses

Three sets of analyses were conducted sequentially on the Decisional Balance for sun protection inventory. The first step tested and confirmed the best-fitting structural model for the Decisional Balance measure. Next, the factorial invariance (stability) of the measurement model was evaluated across multiple population subgroups. All structural equation modeling (SEM) procedures for the first two steps were based on maximum likelihood estimation and performed using EQS 6.1 [22]. The final step determined if the hypothesized functional relationships between each Decisional Balance construct and stages of change would replicate, supporting the known groups and external validity of the measure in this sample [23].

#### 2.3.1. Measurement Structure

Confirmatory factor analysis (CFA) was conducted to establish the best-fitting structural model for the Decisional Balance measure. Two measurement models were compared, including the correlated and uncorrelated two factor models. Model fit was assessed based on several macrofit indices, including the Comparative Fit Index (CFI), Tucker-Lewis Index (TLI), and Root Mean Square Error of Approximation (RMSEA). For the incremental fit indices, CFI and TLI, values of at least .90 indicate an adequate fit, and values above .95 indicate an excellent fit [24–26]. For RMSEA, smaller values indicate a better fit of the model to the data, with values less than .08 considered acceptable and values below .05 indicating a very good fit [25, 26].

#### 2.3.2. Factorial Invariance

Stability of the Decisional Balance measurement structure was assessed across six subgroups defined by gender, racial identity, age, education level, untanned skin color (a proxy for sun reactivity), and stage of change for sun protection [11, 21]. Three levels of invariance were tested, proceeding from the least to the most restrictive: (1) configural invariance with unconstrained factor loadings; (2) pattern identity invariance with factor loadings for like items constrained to be equal across groups; and (3) strong factorial invariance with equal factor loadings and measurement error variances across groups [19, 27–29]. None of the equality constraints were released to achieve a better fit in any of the invariance models assessed. In addition to the model fit indices (CFI, TLI, and RMSEA) described, the difference in CFI (ΔCFI) values between the model and the previous (less restrictive) invariance model was calculated to test the null hypothesis of noninvariance, with a |ΔCFI| value within .01 indicating model invariance [30]. The *χ* ^2^-difference test was also included to assess change in model fit for the nested invariance model comparisons, although this test tends to be overly sensitive to even small differences in fit between models when sample sizes are large [25–27]. Cronbach's coefficient alphas [31] were calculated and used to assess the internal consistency reliability of both Pros and Cons subscales.

#### 2.3.3. Known Groups Validation

A MANOVA, with follow-up ANOVA and Tukey tests, was conducted to examine functional relationships between Decisional Balance (Pros and Cons subscale means in standardized *T*-scores) and the three stage of change groups.

## 3. Results

### 3.1. Analytic Sample

Participants had complete data on the Decisional Balance for Sun Protection measure. Twenty-eight participants (2%) with extreme response patterns on the Decisional Balance measure were deleted, resulting in the final analytic sample of 1336 participants. Sample sizes by category for each of the six population subgroups assessed by invariance testing are presented in Table 1.

### 3.2. Measurement Structure

CFA was conducted on the Decisional Balance measure using the full sample. The measurement model with two uncorrelated factors, consisting of four items each for Pros and Cons (Figure 1), provided a good fit for the data, *χ* ^2^(20) = 138.37, *P* < .001; CFI = .956; TLI = .939; RMSEA = .057 [90% confidence interval = .053, .061]. An alternative model with correlated latent Pros and Cons factors was also assessed and provided a good fit for the data, *χ* ^2^(19) = 138.14, *P* < .001; CFI = .956; TLI = .935; RMSEA = .057 [.052, .061]. The correlation of .016 estimated between the latent Pros and Cons factors was low and not significant, and a *χ* ^2^-difference test comparing the nested correlated and uncorrelated models was also not significant (*χ* ^2^[1] = .23; *P* = .63), indicating that estimating the extra parameter in the correlated model did not improve model fit. The uncorrelated model was therefore retained for parsimony and used for subsequent invariance testing. Baseline models were assessed in each subsample before the model was tested across subsamples. All baseline models fit well (median CFI = .930; median RMSEA = .068).

### 3.3. Factorial Invariance

Multiple-sample CFA was used to examine hierarchical factorial invariance for the two Pros and Cons subscales. The fit indices for the invariance models are summarized in Table 2.

#### 3.3.1. Gender

Sample sizes were adequate to test the models across women (*n* = 842) and men (*n* = 492). Strong factorial invariance provided a good fit for the model for gender (CFI = .946; TLI = .939; RMSEA = .061).

#### 3.3.2. Racial Identity

Sample sizes were adequate for comparing subsamples of participants identified as white (*n* = 1143) or black/African American (*n* = 84). Strong factorial invariance provided a good fit across the two racial identity subsamples (CFI = .945; TLI = .937; RMSEA = .063).

#### 3.3.3. Age

Sample sizes were adequate for five age group subsamples, 18 to 29 years old (*n* = 186), 30 to 39 years old (*n* = 198), 40 to 49 years old (*n* = 346), 50 to 59 years old (*n* = 358), and 60 to 74 years old (*n* = 246). Strong factorial invariance provided a good fit for age (CFI = .935; TLI = .932; RMSEA = .063).

#### 3.3.4. Education

Sample sizes were adequate for three subsamples based on the highest level of education attained, 12 years or less (*n* = 307), 13 to 15 years (*n* = 459), and 16 years or more (*n* = 569). Strong factorial invariance provided a good model fit across education levels (CFI = .940; TLI = .935; RMSEA = .063).

#### 3.3.5. Untanned Skin Color

Untanned skin color was used as a proxy indicator of sun reactivity. Sample sizes were adequate to test the models across subgroups of participants who described their untanned skin color as fair white (*n* = 291), medium white (*n* = 590), and dark white/light brown (*n* = 395). Strong factorial invariance provided a good model fit across skin color (CFI = .941; TLI = .936; RMSEA = .062).

#### 3.3.6. Stage of Change for Sun Protection

Sample sizes were adequate to test the models across participants in precontemplation (*n* = 818), contemplation (*n* = 151), and preparation (*n* = 367). This was the only sequence of nested invariance model comparisons that did not support strong invariance, with a |ΔCFI | >.01 for the comparison between strong invariance and pattern identity invariance. Pattern identity invariance provided an adequate fit across stage (CFI = .934; TLI = .927; RMSEA = .069).

### 3.4. Scale Reliabilities

Strong factorial invariance demonstrated good fit for the cross-sample comparisons across gender, racial identity, age, education level, and skin color. Cronbach's coefficient alphas were therefore calculated for each subscale based on the total sample (see Table 3). The coefficient alphas of .77 for the Pros subscale and .70 for the Cons subscale are consistent with the alphas reported previously [32] and indicate good internal consistency reliability of these two subscales. The factor structure for the eight-item Decisional Balance for sun protection measure is reported with standardized parameter estimates for the entire sample in Figure 1.

### 3.5. Known Groups Validation

A MANOVA was conducted to determine if the Pros and Cons of sun protection differed across the three baseline stage-of-change groups. As predicted [16, 17], there was a significant main effect for stage of change (Wilks' Λ = .83; *F*[4,2664] = 67.26; *P* < .001; multivariate *η* ^2^ = .17). Follow-up ANOVAs and Tukey tests revealed that both the Pros (*F*[2,1333] = 114.59; *P* < .001; *η* ^2^ = .147) and Cons (*F*[2,1333] = 13.91; *P* < .001; *η* ^2^ = .020) differed significantly by stage. Individuals in precontemplation reported significantly lower Pros of sun protection than those in contemplation and preparation. In addition, participants in precontemplation and contemplation reported significantly higher Cons of sun protection than those in preparation. Scale means for the Pros and Cons are shown are Table 4.

## 4. Discussion

This study replicated an uncorrelated two-factor (Pros and Cons) measurement structure for the Decisional Balance for sun protection instrument in a large national sample of adults at risk for sun exposure, confirming the theoretical model from previous studies [12, 17, 21, 32]. Both Pros and Cons subscales showed good internal consistency as seen by alphas of .70 and .77, and the factor loadings for individual items were adequate to excellent (.366 to .865). These results suggest that participants in this sample discriminated between the positive and negative aspects of adopting sun protective behaviors.

The eight-item Decisional Balance for sun protection inventory with two uncorrelated Pros and Cons subscales demonstrated strong factorial invariance in a large population-based sample of adults who did not meet public health criteria for sun protection behavior. This invariance model required that factor loadings and error terms for individual items were constrained to be equal across comparison groups in the model. Strong factorial invariance provided a good fit across gender, racial identity, age, education level, and untanned skin color, based on CFI values around .95 and RMSEA values below .08. The |ΔCFI| values were consistently within the suggested .01 range as each invariance level was assessed hierarchically, demonstrating a high degree of fit for the strong invariance model across the five subgroups. Results of these analyses indicate a consistent relationship between the Pros and Cons factors and the eight items that serve as measured indicators for these two factors.

The pattern identity invariance model demonstrated a reasonably good fit across the stages of change for sun protection, based on fit indices above .90 (CFI > .93; TLI > .92) and RMSEA below .07. This indicates a consistent relationship between the Pros and Cons factors and equivalent loadings for the eight items on these factors across the stages. However, when the item error terms were restricted to be equal in the strong invariance model, |ΔCFI| was considerably over the recommended .01 although the CFI, TLI, and RMSEA values still indicated adequate fit for the model (CFI > .91; TLI > .90). This suggests some slight differences in the measurement model across stage, specifically excess variability in responses on individual items that were not consistent across stage groups. This is perhaps not surprising because it was shown previously that stage of change contributes significantly to variation in the Pros and Cons [16, 17].

Decisional Balance varied across baseline stage-of-change groups, and the overall *η* ^2^ of .17 could be interpreted as a medium multivariate effect size [33, 34]. As expected, participants in the preparation and contemplation stages endorsed the Pros of sun protection more highly compared to those in precontemplation, with *η* ^2^ of .15 representing a large effect of stage of change [34]. Similarly, the Cons of sun protection were rated as less important by participants in preparation compared to those in precontemplation and contemplation. Although the magnitude of the Cons stage effect was small (*η* ^2^ = .02), it should be noted that this baseline sample included only participants who were in the three earliest (out of five possible) stages, because they were recruited to be at risk for sun exposure for the intervention study. It is likely that the reduced variability in the sample attenuated the Cons stage effect compared to including the full range of five stages. Meta-analyses of Decisional Balance suggest that most of the change in Cons occurs between the preparation and maintenance stages [16, 17]. The overall patterns for Pros and Cons across the first three stages of change were consistent with the expected functional relationships based on previous studies [16, 17].

This study has several limitations. First, because there was limited demographic variability in this sample, especially for racial and ethnic identity categories, invariance of the Decisional Balance measurement model could not be assessed across ethnic identity groups. When attempted, the invariance model failed to converge due to too few participants identified as Hispanic (*n* = 56 with complete data). A future sample that is more diverse, with adequate numbers representing additional racial and ethnic groups, would allow more comprehensive assessment of the invariance of this measure beyond white and black racial groups. The sample sizes used in analyses were also unbalanced across racial identity groups, although the invariance models were still indicative of good fit. Second, only the first three (out of five possible) stages of change for sun protection were represented in the baseline sample, which was recruited for an intervention study targeting only at-risk individuals. As described above, this likely restricted the magnitudes of the observed stage effects, especially for the Cons, although the expected cross-sectional differences across the three stages for Decisional Balance were replicated in this sample. Future research is also needed to examine the stability of this measure over time. Third, since this study used a nonclinical, population-based sample, this instrument should undergo additional validation to be utilized with individuals with skin cancer. Finally, the generalizability of the measurement properties of this Decisional Balance instrument is limited to the adult population from which the validation sample was drawn.

## 5. Conclusion

The results of the present study demonstrate that the measurement model for the two uncorrelated factors representing Decisional Balance (Pros and Cons) for sun protection has a consistent relationship across multiple population subgroups, while providing empirical support for the internal and external validity and internal consistency reliability of the measure. The two subscales have demonstrated invariance in factor loadings and measurement error variances across the subgroups assessed and can be used in multiple subgroups, allowing meaningful comparisons to be made across different samples in the target population for these constructs. The cross-sectional relationship between Decisional Balance and the stages of change demonstrated in previous samples was replicated. These findings add to the evidence supporting the use of the Decisional Balance for sun protection inventory in research and intervention.

## Figures and Tables

**Table 1:** Sample size by category for each subgroup.

Subgroup	Category	*N*
Gender	Female	842
	Male	492

Racial identity^a^	White	1143
	Black/African American	84

Ethnicity^b^	Hispanic	56
	Non-Hispanic	1279

Age	18–29 years old	186
	30–39 years old	198
	40–49 years old	346
	50–59 years old	358
	60–75 years old	246

Education level	High school or less (≤12 years)	307
	Some tertiary education (13–15 years)	459
	College graduate or beyond (≥16 years)	569

Untanned skin color	Fair white	291
	Medium white	590
	Dark white/light brown	395

Stage of change for sun protection	Precontemplation	818
	Contemplation	151
	Preparation	367

^a^Not including participants who selected more than one race.

^b^Invariance model could not be assessed across ethnic identity groups due to the low number of participants identified as Hispanic.

**Table 2:** Goodness-of-fit statistics for three nested invariance models.

Model	*χ* ^2^	df	CFI	ΔCFI	TLI	RMSEA	[90% CI]
Gender							
Configural invariance	164.489	40	.952	—	.933	.068	[.058, .079]
Pattern identity invariance	179.778	48	.949	−.003	.941	.064	[.054, .074]
Strong factorial invariance	195.002	56	.946	−.003	.939	.061	[.052, .070]
Racial identity							
Configural invariance	148.451	40	.956	—	.939	.067	[.055, .078]
Pattern identity invariance	164.205	48	.953	−.003	.945	.063	[.052, .073]
Strong factorial invariance	192.406	56	.945	−.008	.937	.063	[.053, .073]
Age							
Configural invariance	240.234	100	.948	—	.927	.073	[.061, .084]
Pattern identity invariance	287.769	132	.942	−.006	.939	.067	[.056, .077]
Strong factorial invariance	339.652	164	.935	−.007	.932	.063	[.054, .073]
Education							
Configural invariance	194.881	60	.951	—	.931	.071	[.060, .082]
Pattern identity invariance	232.137	76	.943	−.008	.937	.068	[.058, .078]
Strong factorial invariance	255.272	92	.940	−.003	.935	.063	[.054, .072]
Untanned skin color							
Configural invariance	190.479	60	.948	—	.928	.072	[.060, .083]
Pattern identity invariance	212.751	76	.946	−.002	.940	.065	[.055, .075]
Strong factorial invariance	242.077	92	.941	−.005	.936	.062	[.052, .071]
Stage of change for sun protection							
Configural invariance	205.704	60	.941	—	.917	.074	[.063, .085]
Pattern identity invariance	238.505	76	.934	−.007	.927	.069	[.059, .079]
Strong factorial invariance	309.411	92	.913	−.021	.906	.073	[.064, .082]

**Table 3:** Summary statistics for Pros and Cons subscales of Decisional Balance (*N* = 1336).

Subscale	Number of items	Mean^a^	Standard deviation	Coefficient alpha	Skewness	Kurtosis
Pros	4	3.35	0.96	.77	−0.29	−0.52
Cons	4	2.92	0.98	.70	0.11	−0.74

^a^Subscale totals divided by number of items before calculating mean and standard deviations.

**Table 4:** Standardized *T*-scores (SD) for Decisional Balance by stage of change (*N* = 1336).

Factor	Stage	*N*	Mean	(SD)	*F*(2,1333)	*η* ^2^	Post hoc Tukey HSD^a^
Pros					114.59^*^	.147	PC < C, PR
	Precontemplation	818	46.99	(9.60)			
	Contemplation	151	53.27	(8.53)			
	Preparation	367	55.36	(8.71)			

Cons					13.91^*^	.020	PC, C > PR
	Precontemplation	818	50.87	(10.11)			
	Contemplation	151	50.92	(9.62)			
	Preparation	367	47.68	(9.55)			

^*^ *P* < .001.

^a^PC indicates precontemplation; C: contemplation; PR: preparation.
